# Short-term cutaneous vasodilatory and thermosensory effects of topical methyl salicylate

**DOI:** 10.3389/fphys.2024.1347196

**Published:** 2024-04-19

**Authors:** Ninja Versteeg, Vanessa Wellauer, Selina Wittenwiler, Dirk Aerenhouts, Peter Clarys, Ron Clijsen

**Affiliations:** ^1^ Rehabilitation and Exercise Science Laboratory (RESlab), Department of Business Economics, Health and Social Care, University of Applied Sciences and Arts of Southern Switzerland, Landquart, Switzerland; ^2^ Department of Movement and Sport Sciences, Vrije Universiteit Brussel, Brussels, Belgium; ^3^ International University of Applied Sciences THIM, Landquart, Switzerland; ^4^ Department of Health, Bern University of Applied Sciences, Berne, Switzerland

**Keywords:** wintergreen oil, blood flow, counterirritation, skin microcirculation, muscle oxygen saturation, skin temperature, vascular physiology, thermal sensation

## Abstract

**Introduction:**

Methyl salicylate, the main compound of wintergreen oil, is widely used in topical applications. However, its vascular and thermosensory effects are not fully understood. The primary aim was to investigate the effects of topical methyl salicylate on skin temperature (T_skin_), skin microcirculation (MC_skin_) and muscle oxygen saturation (SmO_2_) compared to a placebo gel. The secondary aim was to assess thermosensory responses (thermal sensation, thermal comfort) and to explore to which extent these sensations correspond to the physiological responses over time.

**Methods:**

21 healthy women (22.2 ± 2.9 years) participated in this single-blind, randomized controlled trial. Custom-made natural wintergreen oil (12.9%), containing methyl salicylate (>99%) and a placebo gel, 1 g each, were applied simultaneously to two paravertebral skin areas (5 cm × 10 cm, Th4-Th7). T_skin_ (infrared thermal imaging), MC_skin_ (laser speckle contrast imaging) and SmO_2_ (deep tissue oxygenation monitoring) and thermosensation (Likert scales) were assessed at baseline (BL) and at 5-min intervals during a 45 min post-application period (T0-T45).

**Results:**

Both gels caused an initial decrease in T_skin_, with T_skin_(min) at T5 for both methyl salicylate (BL-T5: Δ-3.36°C) and placebo (BL-T5: Δ-3.90°C), followed by a gradual increase (*p* < .001). Methyl salicylate gel resulted in significantly higher T_skin_ than placebo between T5 and T40 (*p* < .05). For methyl salicylate, MC_skin_ increased, with MC_skin_(max) at T5 (BL-T5: Δ88.7%). For placebo, MC_skin_ decreased (BL-T5: Δ-17.5%), with significantly lower values compared to methyl salicylate between T0 and T45 (*p* < .05). Both gels had minimal effects on SmO_2_, with no significant differences between methyl salicylate and placebo (*p* > .05). Thermal sensation responses to topical methyl salicylate ranged from “cool” to “hot”, with more intense sensations reported at T5.

**Discussion:**

The findings indicate that topical methyl salicylate induces short-term cutaneous vasodilation, but it may not enhance skeletal muscle blood flow. This study highlights the complex sensory responses to its application, which may be based on the short-term modulation of thermosensitive transient receptor potential channels.

## 1 Introduction

Methyl salicylate, the main organic compound of natural wintergreen oil, is extracted from the leaves of the wintergreen plant (*lat. Gaultheriae Aetheroleum*) (Ojha et al., 2022). It is commonly used in topical applications such as gels, creams or patches (Yeoh and Goh, 2022). Topical formulations containing methyl salicylate have been shown to be effective in the treatment of muscle strain (Higashi et al., 2010), temporomandibular joint and masseter muscle pain (Lobo et al., 2004). Based on the principle of counterirritation, methyl salicylate has been suggested to have therapeutical effects by triggering irritation or cutaneous pain to mitigate pain of subdermal origin. However, the precise underlying physiological mechanisms are not fully understood (Gammon and Starr, 1941; Green, 1991; Barkin, 2013).

Following application, methyl salicylate rapidly penetrates the skin. In the epidermis and dermis, methyl salicylate undergoes hydrolysis, leading to the formation of active salicylic acid (Martin et al., 2004). Topical salicylates are considered to act primarily as rubefacients (Derry et al., 2014). Accordingly, several studies have recognized methyl salicylate as a vasoactive compound that increases local blood flow and raises skin or tissue temperature (Higashi et al., 2010; Dölen et al., 2015; Petrofsky et al., 2016; Anderson et al., 2017; Wang et al., 2022). Commercially available methyl salicylate products indicated for anti-inflammatory or analgesic purposes mostly contain other active compounds, such as menthol or camphor (Yeoh and Goh, 2022). Given the frequent co-formulation of methyl salicylate with other ingredients, it remains unclear whether the purported vasodilatory effects attributed to methyl salicylate (Higashi et al., 2010; Dölen et al., 2015; Petrofsky et al., 2016; Wang et al., 2022) are due to its individual effects or whether they are primarily mediated by other active compounds. Investigating the vascular effects of methyl salicylate alone may provide insight into its potential mechanisms of action. Moreover, direct penetration of salicylic acid has been shown to be dominant only to a depth of 3–4 mm (Singh and Roberts, 1993) and only small amounts are absorbed systemically (Martin et al., 2004). This suggests that topical methyl salicylate may act as a vasodilator in the skin, but not in the skeletal muscle tissue.

Topical methyl salicylate induces a characteristic thermosensory profile. While methyl salicylate products such as Perskindol^®^ Dolo Gel promise simply a comfortable warming sensation (Verfora, 2022), methyl salicylate induces also sensations of burning, stinging/pricking, and even cold, as opposed to just warmth (Green and Flammer, 1989; Green, 1991). In addition, methyl salicylate enhances the perception of warming and produces hyperalgesia to heating (Green and Flammer, 1989). The distinct thermosensory responses to topical methyl salicylate may be due to pharmacological manipulation of specific thermosensitive nonselective cation transient receptor potential (TRP) channels (Caterina et al., 1997; McKemy et al., 2002; Calixto et al., 2005; Bandell et al., 2007; Levine and Alessandri-Haber, 2007). However, it remains unclear, how the vascular and thermosensory responses to topical methyl salicylate relate over time.

Considering that (1) skin permeation of chemical agents is influenced by age, skin type and sex hormones due to small differences in the chemical composition of the stratum corneum (Souto et al., 2022), and (2) since this is the first study to investigate the vascular effects of methyl salicylate alone, the aim was to examine the effects in a relatively homogenous population. Therefore, the primary aim was to investigate the effects of the topical methyl salicylate on skin temperature (T_skin_), skin microcirculation (MC_skin_) and muscle oxygen saturation (SmO_2_) compared to a placebo gel in healthy women. The secondary aim was to assess thermosensory responses (thermal sensation, thermal comfort) and to explore to which extent these sensations correspond to the physiological responses over time. It was hypothesized that (1) topical methyl salicylate would act as a cutaneous vasoactive compound, resulting in increased MC_skin_ but not SmO_2_; (2) thermal sensations would correspond to changes in T_skin_ and follow a similar time course to the changes observed in MC_skin_ and (3) thermal comfort would be predominantly rated as comfortable.

## 2 Materials and methods

### 2.1 Study design

This study is based on a single-blind, randomized controlled trial. Given potential interindividual variations in skin characteristics may influence skin permeation of topically applied agents (Souto et al., 2022), a within-subject design was chosen. To our knowledge, only data from studies investigating topical methyl salicylate in combination with other active compounds is available (Higashi et al., 2010; Dölen et al., 2015; Petrofsky et al., 2016; Wang et al., 2022). *A priori* sample size calculation for repeated measures ANOVA, within-between interaction (G*power, version 3.1.9.6, Franz Faul, Germany) with an estimated effect size of 0.2, α ≤ .05 and power at 0.8 resulted in a sample size of n = 20. Considering potential dropouts, a total of n = 25 women were recruited from a university population. There was one dropout due to sickness on the day of testing. Three participants had to be excluded due to technical issues (see below), leading to a total sample size of n = 21. The study was approved by the Swiss Ethical Committee of Zurich (KEK-ZH: 2016-01541).

### 2.2 Participants

21 healthy women (age: 22.2 ± 2.9 years, height: 164.9 ± 4.8 cm, body mass: 62.3 ± 8.7 kg, body mass index: 22.9 ± 2.6 kg/m^2^, estimated lower body fat percentage: 31.8 ± 5.5%, subcutaneous adipose tissue layer at the investigated paravertebral skin areas: 3.47 ± 0.58 mm) were included. To be eligible for participation, individuals had to be between 18 and 40 years and have healthy skin conditions. Exclusion criteria were any injury, surgery, or symptoms in rest or under exertion (i.e., swelling, pain, restricted mobility) of the trunk (area between C7 and sacrum including hip on both sides) in the past year, scars or open wounds on the back and medication intake (including over-the-counter medicaments, but excluding contraceptives). Participants were excluded if they were pregnant or breastfeeding. They were excluded if they had polyneuropathy, asthma bronchiale, kidney insufficiency, diabetes mellitus or known allergies to products containing wintergreen oil, menthol or alcohol. Participants were also excluded if they had an altered skin sensation in the area of the back and/or forearm (e.g., numbness, diffuse, tingling, disturbed sensation of hot/cold sensation) or expressed fear of the application or reaction to certain gels such as Perskindol^®^ Classic Gel, Dolo Gel, Cool Gel, Axanova^®^ Hot Gel/Activ Gel, Dolo-X Classic Gel). Participants were informed that a topical application was used.

### 2.3 Gel formulation

The gels used were custom-made by a pharmacy (Apotheke BENU, Landquart, Switzerland). The methyl salicylate gel consisted of natural wintergreen oil (12.9%), hydroxyethyl cellulose (5%), water, methyl parahydroxybenzoate, propyl parahydroxybenzoate, propylene glycol. The natural wintergreen concentration (12.9%) is based on the formulation found in Perskindol^®^ Dolo Gel (Verfora, 2022). Natural wintergreen oil contains at least 99% methyl salicylate. Depending on the geographical location, natural wintergreen oil may also contain other natural compounds such as eugenol (<0.06%) and linalool (<0.03%) (Ojha et al., 2022). Due to their low concentration, any potential impact was considered negligible. The placebo gel, containing the same compounds except for natural wintergreen oil, was used as a control to isolate the specific effects of methyl salicylate.

### 2.4 Screening

After signing a written informed consent form, the participants were screened for eligibility. The screening consisted of (1) a pregnancy test, (2) a questionnaire to evaluate eligibility based on inclusion and exclusion criteria, (3) a product skin tolerance test, and (4) a skin discrimination test. For the product skin tolerance test, the methyl salicylate gel was applied to the volar forearm. This body area was chosen because it is very sensitive and has relatively little hair (Otberg et al., 2004). After application, the skin reaction was observed for 15 min. In case of allergic reaction, the participant was considered not eligible. The skin area on the thoracic spine (Th4-Th7) was chosen as the experimental area because the participants could be blinded to the application of the gels. To control for sensory dysfunction in this area, epicritic and protopathic skin discrimination tests were performed on the skin of the mid-thoracic spine (Th4-L1). Warm-cold discrimination was conducted using test tubes filled with hot and cold water (Odia and Aigbogun, 1988). The skin area on each paravertebral side was touched alternatively, and participants were asked to respond with “hot” or “cold” respectively. Pain perception (sharp-dull discrimination) was tested using a pencil, with the sharp and dull end being randomly applied to the skin. Participants were asked to indicate “sharp” or “dull” (Heutehaus et al., 2021). Vibratory sensation was tested by applying an oscillating tuning fork (128 Hz) to the top of the metatarsophalangeal joint (Hilz et al., 1998; Rowin and Meriggioli, 2007). All participants met the eligibility requirements.

### 2.5 Experimental protocol

Participants were asked not to take any food or drinks (other than water) and to avoid moderate to vigorous physical activity until 2 hours before to the measurements to minimize potential confounding effects on the investigated outcomes. It was advised not to shower or use any body lotion on their back in the morning before the measurement to avoid interference with other topically applied agents. Body height (GPM Stadiometer, Zurich, Switzerland) and body weight were assessed using Tanita body fat scale (TBF-611, Tokyo, Japan), and body mass index (kg/m^2^) was calculated. Lower body fat percentage was estimated using Tanita body fat scale (TBF-611, Tokyo, Japan). Participants were asked to lie in prone position in their underwear. This position should be as comfortable as possible and should not be changed. The skin area of the thoracic spine was rinsed with distilled water (room temperature), using a soft cotton cloth. Two skin areas (5 cm × 10 cm) were marked on each side of the thoracic spine (Th4-Th7) with elastic strips (Leukotape K BSN medical, Hamburg, Germany). Subcutaneous adipose tissue layer at the investigated paravertebral skin areas was determined using ultrasound (MyLab Class C, Esaote, Genoa, Italy) and analyzed with OsiriX Lite software (Pixmeo SARL, Osirix V.8.0.2. Bernex, Switzerland). The allocation of the methyl salicylate and placebo gel applications to the left or right side of the thoracic spine was randomized by the researcher drawing lots (single-blinded). The participants were asked to remain still and to limit their speech to only what was necessary. To avoid any cooling effects through air circulation, all windows and doors were closed and investigators minimized their movements. The environmental conditions in the laboratory were controlled (Voltcraft MT52 digital multimeter, Hirschau, Germany) and kept constant during all experiments (room temperature: 23.3 ± 0.9°C; relative humidity: 39.2 ± 0.8%). During an acclimatization period of 20 min, the participants adapted to laboratory environment, to achieve stable and accurate outcome measurements. To ensure consistent experimental conditions, the methyl salicylate and placebo gel were applied simultaneously. Methyl salicylate and placebo gel were weighed (Kern 770 precision scale, Balingen, Germany), 1 g each, and applied simultaneously paravertebrally by two investigators wearing sterile surgical gloves with a circular motion of the index finger. Consistent application conditions on both sides were maintained by asking the participants to subjectively rate the pressure applied on each side. In addition, the two investigators visually monitored the execution of similar circular motions with the index fingers. Topical methyl salicylate application was expected to have mainly local effects to the site of application (Green and Flammer, 1989). T0 was considered to be the time when both gels had been completely rubbed into the skin. Subsequent measurements were taken at 5-min intervals over a period of 45 min (T5 to T45). The same sequence of assessments was performed at each measurement time point: (1) MC_skin_, (2) T_skin_, (3) thermosensation and (4) SmO_2_. Due to technical issues during data collection three participants had to be excluded from the analysis. During analysis of SmO_2_ data, one outlier was identified with consistently lower values (>1.5 x IQR) in both placebo and methyl salicylate and was excluded from the SmO_2_ dataset. The experimental protocol was otherwise carried out without any deviation.

### 2.6 Outcomes

#### 2.6.1 T_skin_

T_skin_ was recorded using an infrared thermal imaging (FLIR A615 series, Emitec Industrial, Rotkreuz, Switzerland) and data analysis software (FLIR ResearchIR Max) was used to evaluate T_skin_. The emission level of the infrared camera was set to 0.954. The skin areas (5 cm × 10 cm) were manually marked in the software and defined as two separate regions of interest.

#### 2.6.2 MC_skin_

Laser speckle contrast imaging (LSCI) was used to determine MC_skin_ (Abdulhameed et al., 2020), Moor Instruments, Millwey, UK), measuring to a maximum depth of approximately 1 mm in the skin (Limited, 2012). This macroscopic, noncontact device with a high spatial and temporal resolution has been shown to be sensitive for assessing MC_skin_ (Iredahl et al., 2015). The calibration procedure was performed according to the manufacturer’s recommendations the day before the measurements. To minimize the risk of confounding variables, daylight, and other light sources as well as any movement of the LSCI device were diminished. The laser aiming function was used to obtain the optimal distance between the LSCI system and the investigated skin areas. High-resolution LSCI images (576 × 768 pixels), were recorded during a 5s interval (1s/frame, at a frame rate of 25 Hz). The two investigated skin areas (5 cm × 10 cm) were manually marked in the software and defined as two separate regions of interest. The LSCI system uses arbitrary units to express the mean flux in the specified regions of interest which is related to the concentration of circulating erythrocytes in the tissue sample volume, scaled from blue (low perfusion) to red (high perfusion). Images were analyzed using the associated software (MoorFLPI-2 Review V, Moor Instruments, Millwey, UK). Mean flux obtained from the five consecutive LSCI image were averaged and normalized to baseline (BL) values for further analysis.

#### 2.6.3 SmO_2_

Deep tissue oxygenation monitor (moorVMS-NIRS, Moor Instruments, Millwey, UK) was used to assess SmO_2_. Near-infrared spectroscopy (NIRS) technology has been shown to be a non-invasive valid and reliable tool for assessing superficial skeletal muscle oxygenation (Ryan et al., 2013). SmO_2_ measured by NIRS is considered to be an indirect measure of skeletal muscle blood flow (Van Beekvelt et al., 2001b; Ferrari et al., 2004; Barstow, 2019). Two separate probes were placed on each skin area. Each probe consisting of a detector head (containing two photodiodes) and an emitter head (containing two near-infrared LEDs emitting light at 750–850 nm), were separated by a standardized 30 mm probe holder (NPH1-30). The maximum measurement depth of NIRS is thought to be roughly half of the distance between emitter and detector (van Beekvelt et al., 2001a), which would correspond to a depth of 15 mm. Given the measured adipose tissue was 3.47 ± 0.58 mm, NIRS signal therefore reflects the metabolic and hemodynamic changes of the superficial muscle tissue (Ferrari et al., 2004). Adhesive tape (Hypafix, BSNmedical GmbH, Hamburg, Germany) was used to fix the probes to the skin. Oxygenated haemoglobin (oxyHb) and deoxygenated haemoglobin (deoxyHb) were assessed in arbitrary units and SmO_2_ was calculated automatically as oxyHb/(oxyHb + deoxyHb) x 100 (%). Sampling rate was 5 Hz.

#### 2.6.4 Thermosensation

Participants were asked to rate thermal sensation and thermal comfort in each skin area according to the ISO 10551 standard (Fabbri, 2015) using Likert scales (Joshi et al., 2015). By this means, the investigator tapped lightly outside the skin area where the gels were applied. Thermal sensation (“How does this feel like?”) was assessed using the following 9-point Likert scale: 4 = very hot, 3 = hot, 2 = warm, 1 = slightly warm, 0 = neutral, −1 = slightly cool, −2 = cool, −3 = cold, −4 = very cold. This question was slightly adapted from the recommended standardized question (“How are you feeling now?”) to ask specifically about the thermal sensation in the specific skin area. Thermal comfort was assessed (“Do you find this…?”) using a 5-point Likert scale with 0 = comfortable, 1 = slightly uncomfortable, 2 = uncomfortable, 3 = very uncomfortable, 4 = extremely uncomfortable.

### 2.7 Statistical analysis

Statistical analysis was performed using IBM SPSS Statistics V.27 (IBM Corp, Armonk, United States), with a significance level at *p* < .05. Assumption of normality was tested using the Shapiro-Wilk test. Participant’s characteristics were reported descriptively (mean ± SD). Cumulative frequencies were evaluated for thermal sensation and thermal comfort. Repeated measures ANOVA with time (11 time points: BL, T0-T45) and application (methyl salicylate vs. placebo) as within-variables were used for: (1) T_skin_, (2) MC_skin_ and (3) SmO_2_. In case Mauchly’s test indicated that sphericity assumption had been violated, Greenhouse-Geisser correction was used. Estimated effect sizes were calculated using partial eta squared (η_p_^2^), with 0.01, 0.06, and 0.14 being considered as small, medium, and large, respectively (Cohen, 1988). Significant effects were followed up using Bonferroni-adjusted paired t-tests. Figures were created using Prism (9, Graphpad Software, Boston, Massachusetts, United States).

## 3 Results

**FIGURE 1:**
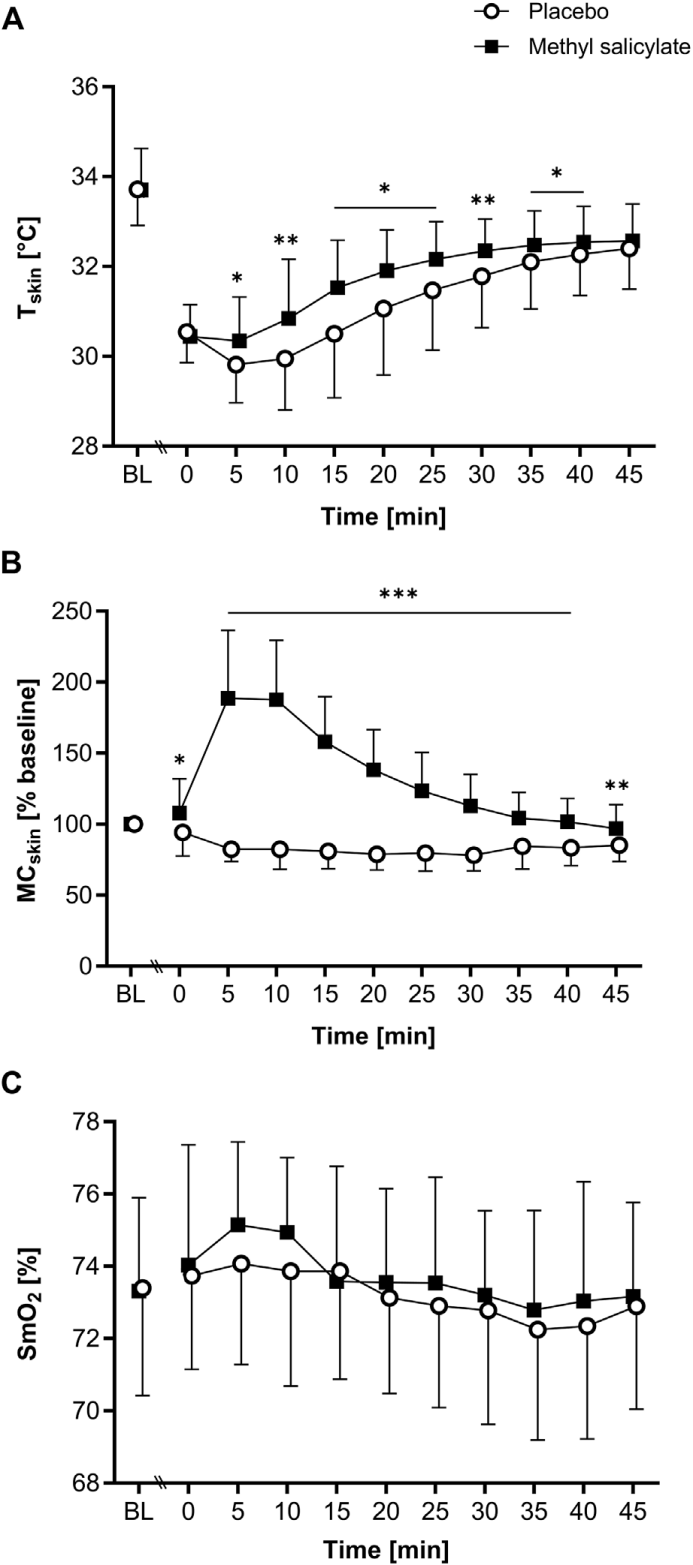
**(A)** Skin temperature (T_skin_), **(B)** skin microcirculation (MC_skin_) and **(C)** muscle oxygen saturation (SmO_2_) for methyl salicylate and placebo at baseline (BL) and during the subsequent 45 min post-application period (T0-T45). MC_skin_ values are normalized to BL (% mean ± SD). The horizontal lines indicate significant differences in methyl salicylate vs. placebo for the indicated time points (***p < .001; **p < .01, *p < .05, Bonferroni-adjusted).

T_skin_, MC_skin_ and SmO_2_ results are shown in Figure 1. Complete repeated measures ANOVA results and *post hoc* pairwise comparisons are presented in Supplementary Tables S1-S3.

### 3.1 T_skin_

A significant main effect of time [F(2.9, 57.9) = 191.1, *p* < .001, η^2^_p_ = 0.905], application [F(1, 20) = 8.22, *p* = .010, η^2^_p_ = 0.291] and interaction [F(2.3, 46.1) = 5.507, *p* = .005, η^2^_p_ = 0.216] was found for T_skin_. Post-hoc pairwise comparisons showed significantly higher T_skin_ for methyl salicylate compared to placebo between T5 and T40 (T5, T15-T25, T35-T40: *p* < .05; T10, T30: *p* < .01). T_skin_ was significantly lower than BL between T0 and T45 for methyl salicylate (T0: Δ-3.26°C; T5: Δ-3.36°C; T10: Δ-2.86°C; T15: Δ-2.17°C; T20: Δ-1.79°C; T25: Δ-1.53°C; T30: Δ-1.35°C; T35: Δ-1.22°C; T40: Δ-1.16°C; T45: Δ-1.13°C, all *p* < .001) and placebo (T0: Δ-3.17°C; T5: Δ-3.90°C; T10: Δ-3.77°C; T15: Δ-3.21°C; T20: Δ-2.65°C; T25: Δ-2.24°C; T30: Δ-1.93°C; T35: Δ-1.61°C; T40: Δ-1.44°C; T45: Δ-1.31°C, all *p* < .001). For both gels, T_skin_(min) was measured at T5, and T_skin_(max) at T45.

### 3.2 MC_skin_

For MC_skin_, a significant main effect of time (F[4.2, 84.4] = 29.389, *p* < .001, η^2^_p_ = 0.595), application [F(1, 20) = 111.745, *p* < .001, η^2^_p_ = 0.848] and interaction [F(2.6, 52.0) = 74.292, *p* < .001, η^2^_p_ = 0.788] was found. Post-hoc pairwise comparisons showed significantly higher MC_skin_ for methyl salicylate compared to placebo between T0 and T45 (T0: *p* < .05, T5-T40: *p* < .001, T45: *p* < .01). For methyl salicylate, MC_skin_ was significantly increased compared to BL between T5 and T25 (T5: Δ88.7%, *p* < .001; T10: Δ87.6%, *p* < .001; T15: Δ58.0%, *p* < .001; T20: Δ38.3%, *p* < .001; T25: Δ23.4%, *p* = .045). For placebo, MC_skin_ was significantly lower compared to BL between T5 and T45 (T5: Δ-17.5%, *p* < .001, T10: Δ-17.6%, *p* < .001; T15: Δ-19.0%, *p* < .001; T20: Δ-21.2%, *p* < .001; T25: Δ-20.2%, *p* < .001; T30: Δ-21.7%, *p* < .001; T35: Δ-15.4%, *p* < .05; T40: Δ-16.6%, *p* < .001; T45: Δ-14.7%, *p* < .001). MC_skin_ (max) was measured at T0 for placebo, and at T5 for methyl salicylate.

### 3.3 SmO_2_

For SmO_2_, a significant main effect of time [F(3.3, 63.4) = 6.51, *p* < .001, η^2^_p_ = 0.255], but no application [F(1, 19) = 1.35, *p* = .26, η^2^_p_ = 0.066] or interaction effect [F(4.7, 89.4) = 1.26, *p* = .29, η^2^_p_ = 0.062] was found. For methyl salicylate, SmO_2_ was significantly higher at T5 (Δ1.83%, *p* = .028) and T10 (Δ1.62%, *p* = .007) compared to BL. For placebo, SmO_2_ did not change significantly compared to BL (*p* > .05).

### 3.4 Thermosensation

**FIGURE 2:**
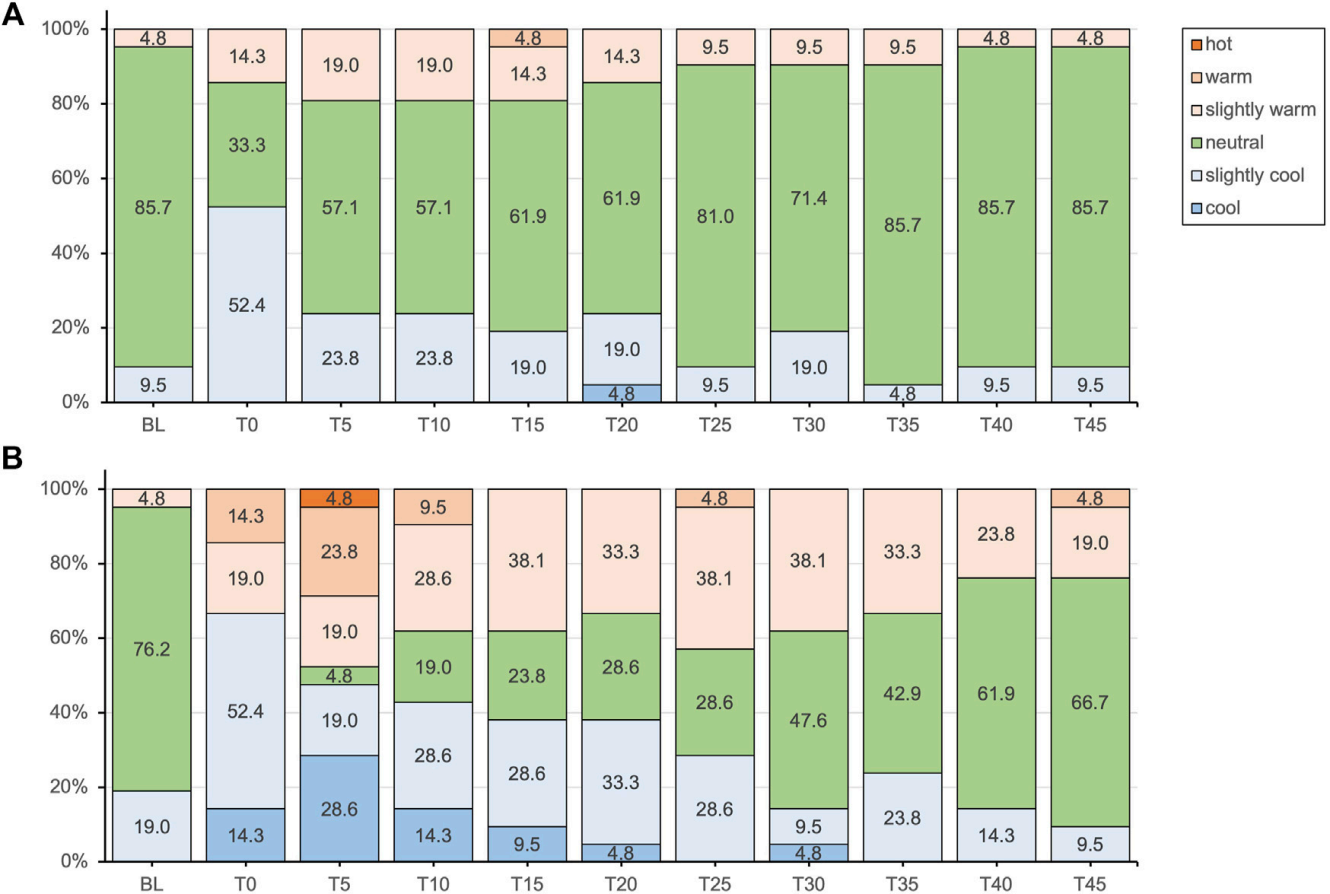
Thermal sensation responses presented as percentages of the total responses for the placebo **(A)** and methyl salicylate **(B)** at baseline (BL) and during the subsequent 45-min post-application period (T0-T45).

**FIGURE 3:**
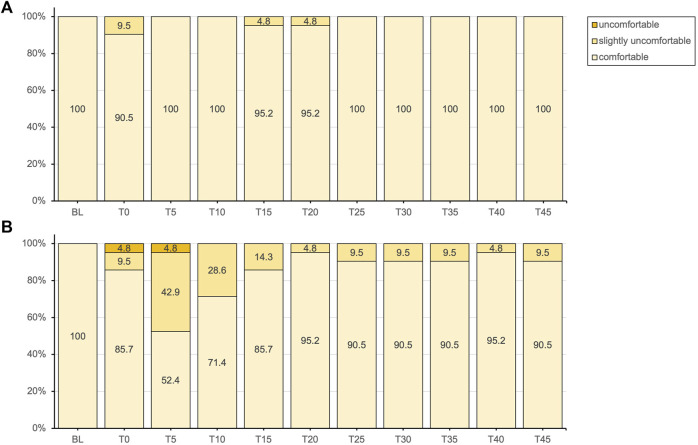
Thermal comfort responses presented as percentages of the total responses for the placebo **(A)** and methyl salicylate **(B)** at baseline (BL) and during the subsequent 45-min post-application period (T0-T45).

Complete thermal sensation and thermal comfort ratings results are displayed in Figures 2, 3 respectively.

## 4 Discussion

This study aimed to investigate the effects of topical methyl salicylate on T_skin_, MC_skin_, and SmO_2_ whilst simultaneously exploring its effects on thermosensation. The findings demonstrate an immediate, non-persisting increase in MC_skin_, but not in SmO_2_. Thermal sensation responses to topical methyl salicylate ranged from “cool” to “hot”, with more intense sensations reported at 5min post-application.

Following application, both gels caused a significant reduction in T_skin_ with minimal temperatures after 5 min (methyl salicylate: Δ-3.36°C, placebo: Δ-3.90°C). These results are consistent with previous studies demonstrating an immediate skin-cooling effect of topical gels in general, with mean/median T_skin_ reductions ranging from −2.1°C to −6.2°C (Hug et al., 2012; Lasanen et al., 2016; Petrofsky et al., 2016; Hunter et al., 2018). For topical menthol applications, it has been found that the cooling effect was not dependent on its concentration (Lasanen et al., 2016) and was found to be comparable to that of a placebo gel (Hunter et al., 2018). Therefore, the immediate cooling effect on tissue/organ temperature was not primarily attributed to the action of the active compound itself, but rather to the evaporation of water and/or alcohol from the skin surface, similar to the mechanism of perspiration (Lasanen et al., 2016; Hunter et al., 2018). The extent of the cooling effect may vary depending on the evaporative properties of the carrier substance, which differ between studies (8.0% (Lasanen et al., 2016) and 35–40% ethanol (Hunter et al., 2018), or isopropyl/propylene glycol (Hug et al., 2012)). In the present study, the carrier substance that included propylene glycol probably resulted in a less pronounced reduction in T_skin_.

Methyl salicylate induced an immediate increase in MC_skin_, peaking at 5 min, and remaining elevated at 10 min post-application, followed by a gradual decline thereafter. In contrast, the placebo gel resulted in consistently reduced MC_skin_ levels throughout the measurement period, with significantly lower levels compared to methyl salicylate. The significant changes observed in MC_skin_ suggest a short-term cutaneous vasodilatory effect of methyl salicylate and provide evidence for the claimed effects of a rubefacient. Many studies have recognized methyl salicylate as a vasoactive ingredient in topical formulations (Higashi et al., 2010; Dölen et al., 2015; Petrofsky et al., 2016; Anderson et al., 2017; Wang et al., 2022). However, given the common co-formulation of methyl salicylate with other ingredients, to date, no evidence was available to confirm the vasodilatory effect of topical formulations containing only this active ingredient.

After the initial drop in T_skin_, both gels resulted in a gradual recovery in T_skin_ over time, with methyl salicylate showing significantly higher temperatures than the placebo gel between 5–40 min post-application. Regulation of local skin blood flow is an essential thermoregulatory mechanism: An increase in MC_skin_ through cutaneous vasodilation generally results in heat dissipation (Tan and Knight, 2018). Therefore, the immediate vasodilatory effect of methyl salicylate may have prevented a further decrease in T_skin_, and subsequently accelerated the increase in T_skin_. Nonetheless, given that after 45 min post-application, T_skin_ was still lower than the BL levels for both gels, indicates a sustained skin-cooling effect of gel evaporation.

While methyl salicylate demonstrated a transient increase in MC_skin_, reflecting altered skin blood flow at a maximum depth of 1 mm (Limited, 2012), it did not induce a concomitant or delayed increase in blood flow within the superficial skeletal muscle (at approximately 15 mm depth (Ferrari et al., 2004)). Changes in SmO_2_ levels over time were minimal for both gels, and no significant difference was found between the gels. This finding suggests that topical methyl salicylate does not enhance skeletal muscle blood flow. Topical products are widely used for the treatment of acute injuries and chronic musculoskeletal conditions (Derry et al., 2014; Derry et al., 2017). Many over-the-counter products are based on natural ingredients including wintergreen oil, menthol, rosemary, capsaicin, or camphor (Morton and Hall, 2002; Barkin, 2013; Kopustinskiene et al., 2022). To date, despite the widespread use of natural compounds and the interest in their therapeutic effects for musculoskeletal conditions, there is limited knowledge of their effects on skeletal muscle blood flow or SmO_2_. One study reported that topical menthol induced blood flow in the quadriceps muscle (Petrofsky et al., 2016). However, the total measurement period (120 min) and the selected interval durations (15 min) may have been too long, considering that menthol is already cleared after 60 min post-application (Craighead et al., 2017). When measured over a measurement period of 50 min, menthol and camphor separately increased SmO_2_ in the forearm, indicating enhanced local perfusion in skin and superficial muscle (Kotaka et al., 2014). Methyl salicylate, menthol and camphor undergo passive diffusion through the epidermis, reach the dermal microvascular networks and are absorbed systemically in detectable but low doses (Martin et al., 2004). Given such low doses, it remains questionable, as to whether, or to what extent, these topical chemicals have vasodilatory effects in the deeper tissues in general (Silva, 2020).

Subjective thermal sensation responses did not entirely align with the objectively measured T_skin_. Following application, the initial decrease in T_skin_ was not clearly perceived with either gel. For methyl salicylate, at T_skin_(min) measured after 5 min post-application, participants tended to experience either cold or warm sensations, with a lower percentage reporting a neutral sensation (4.8% vs. 57.1%). While thermal comfort was predominantly reported as comfortable for both gels, a notable proportion experienced slight thermal discomfort (42.9%) or discomfort (4.8%) after 5 min post-application with methyl salicylate. Over the subsequent measurement period, reaching T_skin_(max) at 45 min, the intensity of these more extreme sensations gradually decreased, with an increasing percentage of participants reporting a neutral sensation similar to placebo. These findings indicate that topical methyl salicylate (12.9%), induced a generally more pronounced and varied thermosensory response within the initial 5 min, which aligns well with the peak in MC_skin_ observed at the same time point. Consistent with these observations, topical methyl salicylate was previously demonstrated to be reliably detectable at concentrations between 3-12%, with a perceived sensation intensity peak between 5–9 min post-application (Green and Flammer, 1989).

Methyl salicylate products such as Perskindol Dolo^®^ are commonly referred to as “hot gels” and are marketed with the implicit or explicit promise of inducing a comfortable and warming sensation (Verfora, 2022). However, the actual thermal sensation responses among participants were more varied and did not clearly evoke a clear cold or warm sensation. This discrepancy supports previous findings that methyl salicylate does not primarily induce a warming sensation (Green and Flammer, 1989) and highlights the complexity of individual sensory responses to topical methyl salicylate. Interestingly, methyl salicylate has been demonstrated to elicit sensation qualities that are independent of temperature, such as burning and stinging/pricking (Green and Flammer, 1989; Green, 1991). Participants were specifically asked to rate thermal sensation using a forced-choice scale, which required them to provide a response even if the sensation experienced was not primarily thermal in nature. This may have introduced a response bias, as the burning sensation evoked by methyl salicylate may have been understood as synonymous with a warm or hot sensation. Therefore, it is important to consider the possibility that participants’ interpretation of the sensations experienced may have been influenced by the presence of burning sensations associated with gel application.

The short-term cutaneous vasodilatory and distinct thermosensory effects of methyl salicylate observed in this study may be based on the short-term activation or inhibition of specific thermosensitive TRP receptors, such as it is suggested for menthol (Macpherson et al., 2006; Craighead et al., 2017; Hunter et al., 2018; Silva, 2020) or camphor (Moqrich et al., 2005; Xu et al., 2005; Selescu et al., 2013; Kotaka et al., 2014). Specifically, methyl salicylate has been shown to have both stimulatory and inhibitory effects on TRPV1 (vanilloid) (Calixto et al., 2005; Ohta et al., 2009), which responds to painful stimuli and heat (>42°C) (Caterina et al., 1997; Bandell et al., 2007), and to activate TRPM8 (melastatin) (Calixto et al., 2005) and TRPA1 (ankyrin) channels (Calixto et al., 2005; Levine and Alessandri-Haber, 2007), which are sensitive to cold temperatures (McKemy et al., 2002) (<25°C and <17°C respectively) (Bandell et al., 2007). In this study, both gels were stored at room temperature (23.3 ± 0.9 °C), which implies that the application of the gels was likely to initially activate TRPM8. Additionally for the methyl salicylate gel, distinct modulation of TRPV1, TRPM8 and TRPA1 (Calixto et al., 2005; Levine and Alessandri-Haber, 2007; Ohta et al., 2009), may have contributed to the varied thermal sensation response. To note, methyl salicylate induced a transient MC_skin_ peak and changes in thermosensation occurred mainly after 5 min post-application. In contrast, the vascular and thermosensory effects of menthol and camphor seem to last remarkably longer (menthol: elevated MC_skin_ between 15–45 min, cooling sensations 5–60 min post-application (Craighead et al., 2017); camphor: elevated MC_skin_ for 50 min and thermal sensations for 30 min respectively (Kotaka et al., 2014)). This suggests that the presumed action of methyl salicylate, menthol and camphor on thermosensitive TRP channels may differ in timing and intensity.

This study aimed to create a realistic therapeutic setting while maintaining a standardized placebo-controlled experiment. As such, the findings are provided considering several limitations and must be interpreted with caution. The protocol ensured consistent use of the same amount of gel, employing a uniform application technique and subjective verification of application pressure and motion. However, the conditions were not completely identical. The (1) variability in the skin penetration endpoint (T0) as well as (2) potential variations in skin humidity and chemical absorption may have introduced slight variability among participants. (3) Moreover, potential interaction effects between thermosensory perception induced by each gel on the left and right side of the spine cannot be excluded. Nevertheless, the within-subject design is likely to outweigh these variations. (4) Thermosensation was determined using slightly adapted standardized subjective ratings for thermal sensation and thermal comfort (Fabbri, 2015). (5) NIRS technology required coverage of the skin areas at each measurement time point, which might have had an occlusive effect of a few seconds on the skin. In light with these methodological limitations, similar studies may benefit from to further standardizing and refining the specific time points related to gel application (e.g., gel exit from the tube, initiation of gel application, completion of gel distribution, and subjective determination of skin penetration endpoint). Furthermore, future studies could consider measuring the temperature of each gel and the fingertips of the assessors applying the gel prior to application. This additional information could enhance the interpretation of T_skin_ results.

Further research is needed to improve our understanding of the effects of natural topical products on deeper laying tissues. Furthermore, it would be valuable to compare the effects of natural wintergreen oil and synthetic methyl salicylate (Ojha et al., 2022). Additionally, alternative delivery forms of methyl salicylate (e.g., patches, sprays) (Yeoh and Goh, 2022), through application during exercise and/or heat exposure (Danon et al., 1986) and/or higher methyl salicylate concentrations could be investigated to optimize skin permeation and improve its efficacy. Specifically for topical methyl salicylate, this study advises the assessment of sensation qualities that are independent of temperature (e.g., burning, stinging, and pricking). Methodologically strong studies are encouraged to thoroughly investigate individual and combined effects of natural compounds, such as menthol and camphor that act on thermosensitive TRP channels. This would contribute to a better understanding of their underlying mechanisms and potential synergistic or additive effects. Given the affordability of topical methyl salicylate, it would be worthwhile to explore its physiological, subjective, and presumed analgesic effects in musculoskeletal conditions. This research could provide valuable insights into its therapeutic potential.

To conclude, this study used advanced non-invasive techniques to investigate the vascular effects of topical methyl salicylate. By including thermosensation assessments, it could be explored how vascular and thermosensory responses relate over time. The findings indicate that topical methyl salicylate induces short-term cutaneous vasodilation, but it may not enhance skeletal muscle blood flow. In addition, this study highlights the complex sensory responses to its application, which may be based on the short-term modulation of thermosensitive TRP channels. This study provides insights for formulators seeking to develop effective topical applications with specific vascular and thermosensory responses, as well as for healthcare professionals advising on their use.

## Data Availability

The original contributions presented in the study are included in the article/Supplementary Material, further inquiries can be directed to the corresponding author.
