# Supplementary material for: Short-term cutaneous vasodilatory and thermosensory effects of topical methyl salicylate
Source: Front Physiol. 2024 Apr 19;15:1347196. doi: 10.3389/fphys.2024.1347196 (PMC11066213; doi:10.3389/fphys.2024.1347196)
Supplement: Supplementary file 1 [file DataSheet1.docx]

Supplementary Material

Short-term Cutaneous Vasodilatory and Thermosensory Effects of Topical Methyl Salicylate

**Ninja Versteeg^1*^, Vanessa Wellauer^1^, Selina Wittenwiler^1^, Dirk Aerenhouts^2^, Peter Clarys^2^, Ron Clijsen^1,2,3,4^**

^1^ Rehabilitation and Exercise Science Laboratory (RESlab), Department of Business Economics, Health and Social Care, University of Applied Sciences and Arts of Southern Switzerland, Landquart, Switzerland

^2^ Department of Movement and Sport Sciences, Vrije Universiteit Brussel, Brussels, Belgium

^3^ International University of Applied Sciences THIM, Landquart, Switzerland

^4^ Department of Health, Bern University of Applied Sciences, Berne, Switzerland

* Correspondence:  
Clijsen Ron

ron.clijsen@supsi.ch

# Supplementary Figures and Tables

**Supplementary Table 1.** Repeated measures analysis of variance (ANOVA) with time (11 time points: BL, T0-T45) and application (methyl salicylate vs. placebo) as within-variables were used for skin temperature (T_skin_), skin microcirculation (MC_skin_) and muscle oxygen saturation (SmO_2_)

| **Outcome** | **Effect** | **Repeated measures ANOVA** | | |
| --- | --- | --- | --- | --- |
|  |  | F (df_XX_, df_error_) = F | p-value | η_p_^2^ |
| T_skin_ | Time | F (2.9, 57.9) = 191.1 | < .001*** | .905 |
|  | Application | F (1, 20) = 8.22 | .010* | .291 |
|  | Interaction | F (2.3, 46.1) = 5.507 | .005** | .216 |
| MC_skin_ | Time | F (4.2, 84.4) = 29.389 | < .001*** | .595 |
|  | Application | F (1, 20) = 111.745 | < .001*** | .848 |
|  | Interaction | F (2.6, 52.0) = 74.292 | < .001*** | .788 |
| SmO_2_ | Time | F (3.3, 63.4) = 6.51 | < .001*** | .255 |
|  | Application | F (1, 19) = 1.35 | .26 (n.s.) | .066 |
|  | Interaction | F (4.7, 89.4) = 1.26 | .29 (n.s.) | .062 |
| ANOVA = analysis of variance, * *p* < .05, ** *p* < .01, *** *p* < .001 | | | | |

**Supplementary Table 2.** Post-hoc pairwise comparisons (Bonferroni adjusted paired t-tests) for skin temperature (T_skin_)

| **Time point** | **Mean±SD placebo** | **Mean±SD methyl salicylate** | **Mean difference**  **Δ placebo – methyl salicylate** | **95% CI of difference** | | **t** | **df** | **p (2-tailed)** | |  |
| --- | --- | --- | --- | --- | --- | --- | --- | --- | --- | --- |
|  |  |  |  | lower | upper |  |  |  |  |  |
| BL | 33.71 ± 0.8 | 33.7 ± 0.93 | 0.0138 | -0.1941 | 0.2218 | 0.139 | 20 | | 0.891 | |
| T0 | 30.54 ± 0.68 | 30.44 ± 0.71 | 0.1000 | -0.1932 | 0.3932 | 0.711 | 20 | | 0.485 | |
| T5 | 29.81 ± 0.85 | 30.34 ± 0.98 | -0.5286 | -0.9801 | -0.0770 | -2.442 | 20 | | 0.024* | |
| T10 | 29.95 ± 1.14 | 30.84 ± 1.32 | -0.8952 | -1.5349 | -0.2556 | -2.920 | 20 | | 0.008** | |
| T15 | 30.5 ± 1.42 | 31.53 ± 1.05 | -1.0286 | -1.7786 | -0.2785 | -2.861 | 20 | | 0.010* | |
| T20 | 31.06 ± 1.48 | 31.91 ± 0.91 | -0.8476 | -1.5385 | -0.1267 | -2.559 | 20 | | 0.019* | |
| T25 | 31.47 ± 1.34 | 32.17 ± 0.83 | -0.6952 | -1.2567 | -0.1338 | -2.583 | 20 | | 0.018* | |
| T30 | 31.78 ± 1.15 | 32.35 ± 0.71 | -0.5667 | -0.9586 | -0.1747 | -3.016 | 20 | | 0.007** | |
| T35 | 32.1 ± 1.04 | 32.48 ± 0.76 | -0.3762 | -0.6694 | -0.0830 | -2.676 | 20 | | 0.015* | |
| T40 | 32.27 ± 0.91 | 32.54 ± 0.8 | -0.2714 | -0.5000 | -0.0429 | -2.477 | 20 | | 0.022* | |
| T45 | 32.4 ± 0.91 | 32.57 ± 0.82 | -0.1667 | -0.3837 | 0.0504 | -1.602 | 20 | | 0.125 | |
| df: degree of freedom, * *p* < .05, ** *p* < .01, *** *p* < .001 | | | | | | | | | | |

**Supplementary Table 3.** Post-hoc pairwise comparisons (Bonferroni adjusted paired t-tests) for skin microcirculation (MC_skin_), values normalized to baseline (BL)

| **Time point** | **Mean±SD placebo** | **Mean±SD methyl salicylate** | **Mean difference**  **Δ placebo – methyl salicylate** | **95% CI of difference** | | **t** | **df** | **p (2-tailed)** | |  |
| --- | --- | --- | --- | --- | --- | --- | --- | --- | --- | --- |
|  |  |  |  | lower | upper |  |  |  |  |  |
| T0 | 94.2 ±16.52 | 107.73 ±24.26 | -13.527 | -23.818 | -3.236 | -2.742 | 20 | | 0.013* | |
| T5 | 82.49 ±8.62 | 188.7 ±47.82 | -106.211 | -127.197 | -85.225 | -10.557 | 20 | | 0.000*** | |
| T10 | 82.44 ±14.3 | 187.6 ±41.83 | -105.157 | -123.579 | -86.734 | -11.907 | 20 | | 0.000*** | |
| T15 | 81.03 ±12.44 | 158.05 ±31.72 | -77.014 | -91.898 | -62.131 | -10.794 | 20 | | 0.000*** | |
| T20 | 78.83 ±11.1 | 138.26 ±28.29 | -59.432 | -71.233 | -47.632 | -10.506 | 20 | | 0.000*** | |
| T25 | 79.76 ±12.75 | 123.37 ±27.19 | -43.605 | -55.741 | -31.468 | -7495 | 20 | | 0.000*** | |
| T30 | 78.28 ±11.05 | 112.91 ±22.26 | -34.625 | -43.949 | -25.301 | -7.746 | 20 | | 0.000*** | |
| T35 | 84.56 ±16.12 | 104.23 ±18.14 | -19.668 | -28.521 | -10.814 | -4.634 | 20 | | 0.000*** | |
| T40 | 83.37 ±12.47 | 101.68 ±16.27 | -18.310 | -26.518 | -10.103 | -4.654 | 20 | | 0.000*** | |
| T45 | 85.32 ±11.54 | 96.96 ±16.66 | -11.645 | -18.833 | -4.458 | -3.380 | 20 | | 0.003** | |
| df: degree of freedom, * *p* < .05, ** *p* < .01, *** *p* < .001 | | | | | | | | | | |
